# Chrono-Exercise Medicine: Why Biological Timing Matters for Human Health

**DOI:** 10.3390/biomedicines14071601

**Published:** 2026-07-17

**Authors:** Stuart J. Hesketh

**Affiliations:** School of Medicine and Dentistry, University of Lancashire, Preston PR1 2HE, UK; shesketh5@lancashire.ac.uk; Tel.: +44-(0)1772-896338

**Keywords:** circadian phenotyping, chronotype, zeitgeber, skeletal muscle clock, exercise prescription, healthy ageing, exercise timing

## Abstract

Exercise medicine has traditionally focused on defining the optimal mode, intensity, duration, and frequency of physical activity required to improve health and performance. However, increasing evidence suggests that biological timing represents an additional and often overlooked determinant of exercise responsiveness. Circadian rhythms regulate numerous physiological processes relevant to exercise adaptation, including metabolism, mitochondrial function, protein turnover, and skeletal muscle function, while exercise itself is increasingly recognised as a potent non-photic zeitgeber capable of influencing circadian organisation. Rather than advocating a universally optimal time-of-day for exercise, this Perspective proposes “chrono-exercise medicine”, as a conceptual framework through which biological timing can be integrated into personalised exercise prescription. It discusses the emerging role of circadian phenotyping, skeletal muscle molecular clocks, and temporal multi-omic approaches for understanding interindividual variability in exercise responses. Together, with key methodological, translational, and clinical challenges that must be addressed to enable biologically informed exercise prescription. It is proposed that the future of precision medicine will depend not only on how exercise is prescribed, but also on when it is prescribed and to whom.

## 1. Introduction

The field of exercise medicine has traditionally focused on defining the optimal mode, intensity, duration, and frequency of physical activity required to improve human health and performance [1,2,3]. While this framework has transformed clinical and sports practice, one biological variable has remained comparatively underexplored: time. Increasing evidence now demonstrates that physiological responses to exercise are strongly influenced by circadian biology, with implications for metabolism, skeletal muscle adaptation, recovery, and long-term health outcomes [4,5]. This emerging area, termed “chrono-exercise medicine”, represents one of the next major advances in personalised exercise prescription.

Circadian rhythms are endogenous ~24-h oscillations that coordinate behaviour and physiology with predictable environmental cycles [6,7]. In mammals, these rhythms are organised by the suprachiasmatic nucleus (SCN) of the hypothalamus, which acts as the central pacemaker and synchronises peripheral tissue clocks throughout the body [8,9]. Importantly, skeletal muscle itself possesses an autonomous molecular clock capable of regulating metabolic and transcriptional processes independent of the SCN [10,11,12]. Core clock genes including BMAL1, CLOCK, PER, and CRY form transcriptional–translational feedback loops [6] that coordinate daily rhythms in mitochondrial metabolism [13], gene expression [14], substrate utilization [15], protein turnover [16], and contractile function [17,18].

In recent years, studies in both rodents and humans have demonstrated clear time-of-day variation in exercise capacity and adaptation [5,19]. Human exercise performance often peaks in the late afternoon or early evening, coinciding with elevated body temperature, enhanced neuromuscular efficiency, and altered substrate metabolism [5]. Similarly, rodent studies have shown that exercise timing can substantially alter the transcriptional and metabolic response to training [19,20,21]. These findings suggest that exercise acts not only as a physiological stimulus, but also as a potent zeitgeber capable of influencing peripheral circadian organization [22].

**Figure 1:**
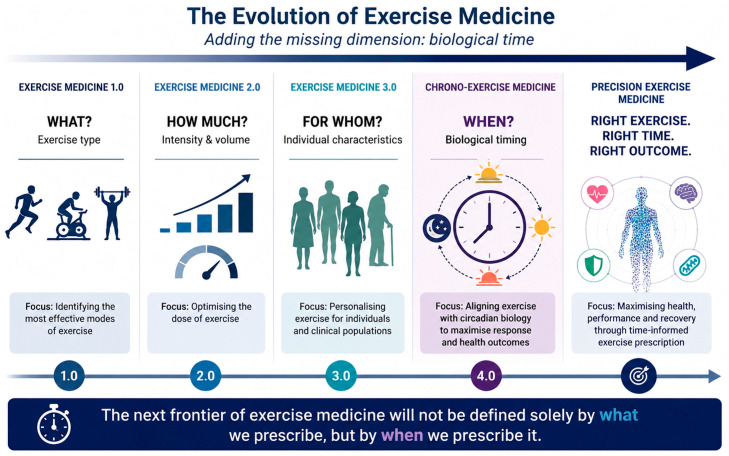
Conceptual framework of Chrono-Exercise Medicine.

Recent reviews have comprehensively summarised the molecular mechanisms underpinning circadian regulation of exercise physiology, the bidirectional interactions between exercise and the circadian clock, and the influence of exercise timing on metabolic health and human performance [5,23,24]. Collectively, these works have substantially advanced our understanding of how biological timing influences physiological responses to exercise. Rather than providing another narrative synthesis of this rapidly expanding evidence base, the present *Perspective* proposes “Chrono-exercise medicine” as an overarching conceptual framework that integrates circadian phenotyping, molecular profiling, methodological standardisation, and precision exercise prescription within a clinically translatable model (Figure 1). By shifting the focus from whether biological timing influences exercise responses to how circadian biology can be systematically incorporated into personalised healthcare, this Perspective seeks to define future priorities for research and clinical implementation, by the development of biologically individualised exercise medicine.

Despite this growing evidence base, current exercise guidelines rarely account for individual circadian characteristics or even consider biological timing. This may partially explain why substantial interindividual variability exists in training responsiveness and exercise-mediated health outcomes [25,26,27,28]. Individuals differ markedly in chronotype, sleep behaviour, circadian alignment, occupational schedules, and habitual activity timing [28,29,30]. Consequently, identical exercise interventions performed at different biological times may elicit divergent physiological responses between individuals.

Importantly, biological timing is dynamic and varies considerably between individuals across the lifespan. Chronotype, circadian phase, and behavioural rhythms change with age, while circadian disruption becomes increasingly prevalent in older adults owing to reduced rhythm amplitude, altered sleep architecture, and diminished responsiveness to environmental time cues [28,31,32,33]. Furthermore, growing evidence demonstrates that biological sex influences multiple aspects of circadian physiology, including circadian timing, resilience to circadian disruption, and susceptibility to metabolic dysfunction [34]. These differences are likely to contribute to variability in exercise responses and further highlight the limitations of adopting a universal approach to exercise timing. Rather than seeking a single “optimal” time to exercise, chrono-exercise medicine should instead aim to identify the most appropriate exercise timing for an individual based upon their circadian phenotype, age, sex, lifestyle, and underlying health status [31,34]. Such an approach is fundamental to the development of truly personalised exercise prescription.

## 2. Circadian Phenotyping: A Tiered Approach

At present, the available evidence does not support the existence of a universally optimal time-of-day for exercise. Instead, the healthiest and most effective time to exercise is likely to be the one that best aligns with an individual’s circadian biology while maximising long-term adherence, safety, and therapeutic benefit. Consequently, the central objective of chrono-exercise medicine is not to identify a single “best” time to exercise, but to determine the most appropriate timing for each individual according to their biological, behavioural, and clinical characteristics (Figure 1). The concept of circadian phenotyping therefore becomes increasingly important.

Circadian phenotyping refers to the comprehensive assessment of an individual’s biological timing characteristics through the integration of behavioural, physiological, and molecular measures that collectively define circadian phase and temporal phenotype. At its most accessible level, assessment may incorporate validated chronotype questionnaires, including the Morningness–Eveningness Questionnaire and Munich Chronotype Questionnaire, together with sleep diaries, wearable technologies, and habitual activity monitoring to characterise an individual’s daily behavioural rhythms [31,34,35]. Within research settings, this framework can be expanded through objective assessment of sleep–wake timing using actigraphy, quantification of environmental light exposure, meal timing, physical activity patterns, and other behavioural factors known to influence circadian physiology [23,35,36,37]. At the highest level of precision, emerging approaches incorporating dim-light melatonin onset (DLMO), endocrine biomarkers, transcriptomic, proteomic, phosphoproteomic, metabolomic, and other multi-omic technologies offer the opportunity to define biological timing with substantially greater mechanistic resolution [16,24,38]. Importantly, this tiered framework provides a scalable approach through which circadian phenotyping can be implemented across both research and clinical practice, allowing exercise prescription to evolve from population-based recommendations towards biologically informed interventions tailored to an individual’s circadian phenotype. As molecular technologies become increasingly accessible, comprehensive circadian phenotyping is likely to become the foundation upon which precision exercise medicine is built.

Such approaches may become particularly relevant in clinical populations. Modern society increasingly exposes individuals to circadian disruption through shift work, artificial light exposure, sleep restriction, and irregular behavioural patterns. Circadian misalignment has been associated with obesity, insulin resistance, cardiovascular disease, sarcopenia, and impaired metabolic health [4]. Exercise is already prescribed as a first-line therapy for many chronic diseases. However, current clinical guidelines rarely consider the timing of exercise relative to an individual’s circadian biology. This represents a significant gap in personalised healthcare, particularly given growing evidence that biological timing influences metabolic regulation, exercise responsiveness, and physiological adaptation [5,20,24].

Importantly, the relationship between exercise and circadian biology is increasingly recognised as bidirectional. While early chrono-exercise research primarily sought to determine how circadian rhythms influence exercise performance and training adaptation, recent work has demonstrated that appropriately timed exercise can itself modify circadian organisation through phase-shifting of behavioural rhythms, entrainment of peripheral molecular clocks, and alterations in clock-controlled metabolic pathways [19,21,22,23,24,39]. This shift in perspective fundamentally expands the scope of chrono-exercise medicine, from identifying when exercise should be performed for each individual, to understanding the molecular mechanisms through which appropriately timed exercise can restore circadian organisation and improve human health.

## 3. Skeletal Muscle as the Molecular Interface

Exercise is increasingly recognised not only as a behavioural output of circadian regulation, but also as a potent non-photic zeitgeber capable of influencing both central and peripheral clocks [22,39]. Experimental studies in both animals and humans demonstrate that appropriately timed exercise can induce phase shifts in circadian rhythms, alter clock gene expression, and modify behavioural timing patterns [22,39,40]. These observations suggest that exercise may represent one of the most powerful behavioural interventions available for modulating circadian organisation. Consequently, exercise should perhaps be viewed not only as a treatment for metabolic disease and physical dysfunction, but also as a therapeutic strategy capable of targeting circadian disruption itself.

The therapeutic implications of this concept extend beyond exercise performance and adaptation. Modern lifestyles are increasingly characterised by circadian disruption arising from shift work, irregular sleep schedules, artificial light exposure, and reduced behavioural regularity. Such disturbances are associated with elevated risk of metabolic disease, cardiovascular dysfunction, cognitive impairment, and accelerated biological ageing [41,42]. Given that exercise is already widely prescribed to improve health across these conditions, an important unanswered question is whether appropriately timed exercise can simultaneously enhance physiological adaptation and strengthen circadian organisation. Addressing this question may help establish chrono-exercise medicine as a framework through which exercise is used not only to improve health outcomes, but also to restore temporal biological organisation itself (Figure 1).

Among the tissues through which these interactions are likely to be mediated, skeletal muscle occupies a uniquely important position as the principal biological interface between circadian biology and exercise. Beyond its fundamental role in locomotion, skeletal muscle is the largest insulin-sensitive organ in the body and a major regulator of systemic glucose disposal, energy expenditure, metabolic flexibility, and whole-body metabolic homeostasis [43]. Importantly, its intrinsic molecular clock coordinates rhythmic metabolic and physiological processes that directly influence exercise adaptation [14,44], positioning skeletal muscle at the centre of the chrono-exercise medicine framework. Disruption of this intrinsic clock in skeletal muscle specifically has been associated with impaired insulin sensitivity, mitochondrial dysfunction, altered substrate metabolism, and reduced exercise capacity, supporting the concept that skeletal muscle serves not only as a target of circadian regulation, but also as a key mediator through which biological timing influences physiological adaptation [45,46,47,48,49].

Despite the central role of skeletal muscle, our mechanistic understanding of chrono-exercise biology remains surprisingly limited. To date, many human chrono-exercise studies have relied upon isolated biomarkers or small candidate-gene approaches, which capture only a limited fraction of the complex molecular networks governing circadian regulation and exercise adaptation [20,23,24,25,38]. Furthermore, most investigations have characterised molecular responses at only one or two discrete time points, providing little insight into the dynamic temporal architecture of circadian physiology. Such approaches are inherently unable to resolve the coordinated oscillations in transcription, protein abundance, post-translational signalling, and metabolism that underpin circadian adaptation. Fully elucidating the molecular basis of chrono-exercise medicine will therefore require systems-level investigation using integrated temporal multi-omic approaches capable of capturing biological responses across the circadian cycle.

The next generation of chrono-exercise medicine research should therefore move beyond simply incorporating multi-omic technologies and instead embrace temporally resolved molecular phenotyping. Rather than relying on single pre- and post-exercise biopsies or isolated molecular endpoints, future human studies should combine repeated skeletal muscle sampling across the circadian cycle with integrated transcriptomic, proteomic, phosphoproteomic, and metabolomic analyses. Coupled with comprehensive circadian phenotyping, wearable-derived behavioural monitoring, and rigorous control of environmental and behavioural zeitgebers, these approaches would allow the temporal architecture of human exercise adaptation to be characterised at an unprecedented level of resolution. Such study designs have the potential to identify molecular signatures of circadian alignment, reveal time-dependent regulatory networks governing adaptation and recovery, and distinguish individuals who are most likely to benefit from biologically aligned exercise interventions. Ultimately, achieving high temporal resolution through integrated multi-omic profiling will transform chrono-exercise medicine from a largely descriptive discipline into a predictive and mechanistically informed science, providing the biological foundation upon which truly personalised exercise prescription can be developed.

## 4. From Concept to Clinical Implementation

The successful translation of chrono-exercise medicine into clinical practice will require a fundamental shift in how biological timing is incorporated into exercise prescription. Rather than seeking a universally optimal time of day for exercise, future exercise interventions should integrate circadian biology alongside established determinants including age, sex, disease status, training history, lifestyle, and individual therapeutic goals. Recent large-scale epidemiological studies have highlighted the complexity of this relationship. Accelerometer-derived analyses from the UK Biobank have demonstrated that the association between physical activity timing and cardiovascular disease risk differs according to when habitual activity is performed, independent of total physical activity volume [50]. Likewise, prospective cohort data indicate that while moderate-to-vigorous physical activity confers health benefits throughout the day, specific timing patterns are associated with greater reductions in all-cause and cardiovascular mortality, particularly in older adults and individuals with pre-existing cardiovascular disease [51]. These data emphasise that biological timing represents an additional dimension of precision medicine rather than a replacement for current exercise recommendations and reinforce the concept that the most appropriate time to exercise is likely to differ between individuals rather than being universally prescribed.

This principle has important implications across diverse clinical and performance settings. In healthy ageing, exercise timing may be leveraged to preserve skeletal muscle function, improve metabolic resilience, and reinforce declining circadian organization [4,31,47]. In individuals with obesity, type 2 diabetes, or cardiovascular disease, appropriately timed exercise may complement endogenous rhythms in glucose metabolism, insulin sensitivity, and vascular physiology to enhance therapeutic efficacy [4,52,53]. Conversely, within athletic populations, exercise timing strategies may be employed to optimise adaptation, competition readiness, or circadian re-entrainment following travel or shift in competition schedules [5,54,55]. Collectively, emerging evidence suggests that exercise timing should not be viewed as a universal recommendation, but rather as a context-dependent component of personalised healthcare that is tailored according to the physiological characteristics and clinical objectives of each individual [23,56,57].

Realising this vision will require considerably greater methodological standardisation than currently exists within the field. Recent recommendations have highlighted the need to systematically characterise and report chronotype, habitual sleep–wake behaviour, prior physical activity, nutritional timing, environmental light exposure, menstrual status where appropriate, and other behavioural factors known to influence circadian physiology [36]. The recently proposed “Standardised Approach” for chronobiology and exercise research provides an important methodological framework for both researchers and clinicians by encouraging rigorous assessment and transparent reporting of the principal variables influencing biological timing. Such an approach will improve reproducibility, facilitate meaningful comparison between studies, and accelerate the translation of chrono-exercise medicine from proof-of-concept research towards evidence-based clinical implementation [36].

Clinical implementation is also unlikely to depend upon a single biomarker or diagnostic tests. Instead, chrono-exercise medicine will likely develop through a tiered approach to circadian phenotyping. Initial implementation may rely upon practical tools including chronotype questionnaires, sleep diaries, wearable technologies, and behavioural assessments to optimise exercise scheduling. As molecular technologies become increasingly accessible, these approaches may be complemented by hormonal biomarkers together with transcriptomic, proteomic, phosphoproteomic, and metabolomic profiling capable of defining an individual’s biological timing with substantially greater precision. Such a framework would enable exercise prescription to evolve beyond recommendations based solely upon exercise modality, intensity, and volume towards interventions tailored according to an individual’s circadian phenotype, physiological characteristics, and clinical objectives.

## 5. Current Challenges

Despite considerable progress, several fundamental challenges must now be addressed before chrono-exercise medicine can become routine clinical practice. Future advances will depend upon developing robust and clinically accessible approaches to circadian phenotyping, identifying reliable molecular biomarkers of biological timing and exercise responsiveness, determining how biological timing influences long-term adaptation across diverse populations, establishing standardised methodological frameworks that improve reproducibility, and translating these discoveries into pragmatic exercise interventions that meaningfully improve health outcomes. Addressing these challenges raises several important questions. Can exercise be prescribed not only to improve health, but also to restore circadian organisation in individuals experiencing chronic circadian disruption? Which molecular signatures most accurately define biological timing within metabolically active tissues? Can temporal multi-omic profiling identify individuals most likely to benefit from biologically aligned exercise interventions? And ultimately, can circadian-informed exercise prescription deliver clinically meaningful benefits beyond those achieved through conventional exercise programmes? Answering these questions will require coordinated interdisciplinary research integrating circadian biology, exercise physiology, systems biology, bioinformatics, and clinical medicine.

Future studies must therefore move beyond simplistic “morning versus evening” comparisons and instead adopt rigorous chronobiological study designs capable of capturing the complexity of human temporal physiology. Biological timing should not be considered a binary variable, but rather a dynamic and multidimensional characteristic influenced by chronotype, habitual sleep–wake behaviour, behavioural regularity, nutritional timing, environmental light exposure, and circadian phase itself. Consequently, future investigations should incorporate comprehensive circadian phenotyping alongside repeated assessments across multiple time points throughout the waking day, rather than relying solely on comparisons between two arbitrarily selected testing windows. Such approaches will be essential for distinguishing true circadian influences from behavioural, environmental, and methodological confounders that have contributed to heterogeneity within the existing literature.

Equally important will be the development of longitudinal intervention studies designed to determine whether chronobiologically aligned exercise training can improve clinically meaningful outcomes in metabolic disease, healthy ageing, rehabilitation, and performance settings. Achieving this objective will require integration of molecular profiling, wearable technologies, and advanced circadian assessment tools capable of identifying both an individual’s circadian phase and their responsiveness to exercise at different biological times. Ultimately, the future of chrono-exercise medicine lies not in determining whether morning or evening exercise is universally superior, but in understanding when exercise should be prescribed for a given individual, under specific physiological conditions, to maximise adaptation, recovery, and long-term health outcomes.

## 6. Concluding Remarks

The integration of circadian biology and exercise physiology represents one of the most promising opportunities for the future of personalised medicine. As molecular technologies, circadian phenotyping, and methodological standardisation continue to evolve, exercise prescription can move beyond population-based recommendations towards biologically individualised interventions. We therefore define chrono-exercise medicine as the integration of circadian biology into exercise prescription through the assessment of biological timing and the strategic prescription of exercise timing to optimise physiological adaptation, health, and disease management. The central question is therefore no longer whether exercise works as medicine, but whether medicine itself should begin to consider when exercise is prescribed. The next generation of exercise medicine may ultimately be defined not only by how much exercise we prescribe, but by when, for whom, and why we prescribe it.

## Data Availability

No new data were created or analyzed in this study. Data sharing is not applicable to this article.

## Abbreviations

The following abbreviations are used in this manuscript:

SCN	Suprachiasmatic nucleus
BMAL1	Brain and Muscle ARNT-like
CLOCK	Circadian Locomotor Output Cycles Kaput
PER	Period
CRY	Cryptochrome
